# Acute fatal chest pain: optimized procedure in emergency department

**DOI:** 10.1186/1471-227X-13-S1-S4

**Published:** 2013-07-04

**Authors:** Yang Yang, Wei Zhang, Ming Peng, Lianying Tong, Shouyu Lin

**Affiliations:** 1 Department of emergency, Fuzhou general hospital of Nanjing military command, Fuzhou 350025, China

## Abstract

**Objective:**

To explore the diagnostic procedure of acute fatal chest pain in emergency department (ED) in order to decrease the misdiagnosis rate and shorten the definite time to diagnosis. The ultimate aim is to rescue the patients timely and effectively.

**Methods:**

Three hundreds and two patients (56.9±11.8 Years, 72% men) complained with acute chest pain and chest distress presenting to our ED were recruited. They were divided into two groups according to visiting time (Group I: from October 2010 to March 2011, Group II: from October 2011 to March 2012). The misdiagnosis rate, definite time for diagnosis and medical expense were analyzed. Patients of Group I were diagnosed by initial doctors who made their diagnosis according to personal experience in outpatient service or rescue room in ED. While patients of Group II were all admitted to rescue room and were diagnosed and rescued according to the acute chest pain screening flow-process diagram. Differences inter-group was compared.

**Results:**

The misdiagnosis rate of fatal chest pain in Group I and Group II was 6.8% and 0% respectively, and there was statistic difference (P=0.000). The definite time to diagnosis was 65.3 min and 40.1 min in control and Group II respectively, the difference had statistic significance (P=0.000). And the mean cost for treatment was 787.5/124.5 ¥/$ and 905.5/143.2 ¥/$ respectively, and there was statistic difference too (P=0.012).

**Conclusion:**

Treating emergency patients with acute chest pain according to the acute chest pain screening flow-process diagram in rescue room will decrease misdiagnosis apparently, and it can also shorten the definite time to correct diagnosis. It has a remarkable positive role in rescuing patients with acute chest pain timely and effectively.

## Introduction

Chest pain is a common urgent condition in emergency department (ED). And fatal chest pain is a more serious condition threatening the lives of patients and security of medical treatment. The definite time to correct diagnosis of fatal chest pain is an important factor for treatment and prognosis [1]. However, phenomena with misdiagnosis or too long time to definite diagnosis of fatal chest pain could often be seen, and thus the appropriate treatment was delayed. How to decrease the misdiagnosis rate and shorten the definite time to diagnosis is a challenge for emergency doctors and those who must face it. Our ED has taken the advantage of the advanced rescue facilities and the quality of staff since June 2011. We rescued patients with acute chest pain in standardization according to acute chest pain screening flow-process diagram and acquired satisfactory results.

## Patients and methods

### Patients

Three hundreds and two patients complained of acute chest pain and chest distress aged from 19 to 84 years (56.9±11.8 Years, 72% men) were recruited to our study. The patients were divided into two groups according to the visiting time to ED. The patients visiting from October 2010 to March 2011 were enrolled in Group I, and Group II included patients visiting from October 2011 to March 2012. The misdiagnosis rate, definite time for diagnosis and medical expense were analyzed.

### Methods

**Figure 1:**
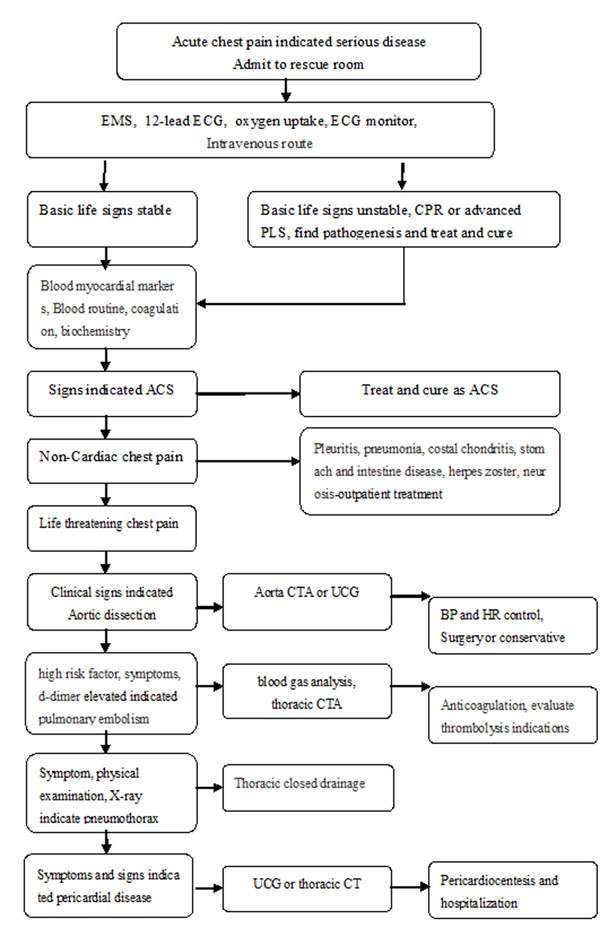
Screening of acute chest pain in emergency department

The patients of Group I were triaged by the responsible nurse to outpatient service or rescue room of ED. And they were diagnosed by initial doctors according to personal judgment and experience. While patients of Group II were all enrolled in rescue room and were diagnosed and rescued according to the acute chest pain screening flow-process diagram (Figure 1). The diseases associated with fatal chest pain include acute myocardial infarction, unstable angina, pulmonary embolism, aortic dissection, pneumothorax and cardiac tamponade. Misdiagnosis includes error diagnosis, delay diagnosis (beyond two hours) and missed diagnosis[2]. The definite time to diagnosis means time from patient’s visiting to getting definite diagnosis.

### Statistics processing

SPSS 13.0 software was used for data management and analysis. Measurement data was described as . Difference inter-group was compared with t-test. Count data was analysed with nonparametric tests, P<0.05 was considered to have statistic difference.

## Results

In 147 patients with acute chest pain in Group I, 137 were definitely diagnosed as fatal acute chest pain. And 10 were misdiagnosed. The misdiagnosis rate was 8.53%. The mean definite time to diagnosis was 65.3±35.0min. All 155 patients with acute chest pain in Group II, were definitely diagnosed as fatal acute chest pain. 0 was misdiagnosed. Misdiagnosis rate was 0%. The mean definite time to diagnosis was 40.1±12.7min (Table 1).

**Table 1:** Comparison of misdiagnosis rate, definite time to diagnosis and medical expenses between the two groups

	Number	Definite diagnosis	Misdiagnosis rate (%)	Time to definite diagnosis (min)	Medical expenses (¥/$)
Group I Group II P	147 155	137 155	8.53 0 0.000	65.3±35.0 40.1±12.7 0.000	787.5/124.5±351.0/55.5 905.5/143.2±426.1/67.4 0.012

## Discussion

Acute chest pain is a very serious emergency that threatened patient’s lives. Make a definite diagnosis as soon as possible and start certainty therapy is very critical. The main factors that delayed diagnosis and treatment of this kind of patients include insufficient cognition by oneself, time delays before visiting and diagnosis and management delayed in the hospital [3]. And the third factor is the medical part that can be improved fast as possible.

In the past, the hospital pattern of diagnosis and treatment presented as assessment or previewing patients with acute chest pain by physicians depending on their personal medical expertise. This kind of pattern had multiple disadvantages. Firstly, medical resources could not be distributed effectively and reasonably. The amount of emergency patients in hospitals of class three grades A increased rapidly with the social development and the concept transition. Sometimes, there were too many patients lining up in apex time. Emergency doctors had no time to care for some patients who really needed diagnosis and cure in precedence. And acute chest pain is one of the preferential diseases. If these patients were passive and then too much time was lost in examination for first things first, that would cause the delay of diagnosis and cure [4]. Secondly, triage of outpatients depended on the experience of charged nurse. Since her or different physicians’ experience and level were distinct, that would cause objective triage and incorrect evaluation. That resulted in failing to discover and diagnose some insidious fatal chest pain.

To aim directly at the above issues, we adopted two major measures to solve them. First, all the patients complained with chest pain and chest distress were admitted to rescue room for diagnosis and cure. The consummate monitor and emergency equipments ensured the compact diagnose and examination time, which should be controlled better. And the misdiagnosis rate of fatal chest pain was thus decreased [5]. Second, the procedure of diagnosis and cure was carrying out according to the acute chest pain screening flow-process diagram. This procedure avoided the experience and level difference between doctors, and the misdiagnosis rate of fatal chest pain was reduced. Through the practice of the two measures, we found the misdiagnosis rate of fatal chest pain was decreased, and the definite time to diagnosis was shortened obviously. We believe that the definite time to diagnosis is more important in rescue the patient’s life. Earlier period diagnosis and definitive treatment can improve the prognosis and reduce the complication apparently, and also lessen the patients’ economic burden of continued treatment. We compared the probably increase of patients in rescue room and the probably increase of medical cost. We found that the passage was not blocked because of the reasonable evaluation procedure, although the patients were increased. The mean residence time in rescue room was no more than half an hour. In addition, it was true that the medical costs of all the patients with chest pain were increased slightly before definite diagnosis, but it was not increased obviously compared with the annum emergency cost of all the patients. Consideration of the benefit of continued cost decrease brought by early diagnosis and improvement of prognosis, the slightly increase of the emergency expenses is acceptable.

To sum up, we think it is important to start the rescue procedure of acute chest pain when patients complain of high-risk symptoms. And the acute chest pain screening flow-process diagram should be followed in diagnosing and rescuing patients in rescue room.

## Declarations

This article has been published as part of *BMC Emergency Medicine* Volume 13 Supplement 1, 2013: Proceedings of the 2012 Emergency Medicine Annual Congress. The full contents of the supplement are available online at http://www.biomedcentral.com/bmcemergmed/supplements/13/S1.

The publication costs for this article was funded by Fuzhou general hospital of Nanjing military command, Fuzhou 350025, China.
